# ﻿The genus *Oligonychus* Berlese (Acari, Prostigmata, Tetranychidae): taxonomic assessment and a key to subgenera, species groups, and subgroups

**DOI:** 10.3897/zookeys.1079.75175

**Published:** 2021-12-22

**Authors:** Hafiz Muhammad Saqib Mushtaq, Fahad Jaber Alatawi, Muhammad Kamran, Carlos Holger Wenzel Flechtmann

**Affiliations:** 1 Acarology Laboratory, Department of Plant Protection, College of Food and Agriculture Sciences, King Saud University, P.O. Box No. 2460, Riyadh 11451, Saudi Arabia King Saud University Riyadh Saudi Arabia; 2 Departamento de Entomologia e Acarologia, Escola Superior de Agricultura “Luiz de Queiroz”, Universidade de São Paulo, 13418–900, Piracicaba, São Paulo, Brazil Universidade de São Paulo São Paulo Brazil

**Keywords:** Morphology, species complex, species identification, species inquirenda, spider mite, taxonomy

## Abstract

A comprehensive taxonomic assessment of the most agriculturally important and highly diverse spider mite genus, *Oligonychus* Berlese (Acari: Tetranychidae) was performed. The sub-generic division, species groups, doubtful species, species complexes and the interpretation of a key generic character are discussed. Based on the orientation of the male aedeagus, only two subgenera, namely *Oligonychus* Berlese (aedeagus downturned) and *Reckiella* Tuttle & Baker (aedeagus upturned), are valid in the genus *Oligonychus*. The subgenera *Homonychus* Wainstein, *Metatetranychoides* Wainstein, and *Wainsteiniella* Tuttle & Baker are considered to be synonyms of the subgenus Oligonychus, whereas the subgenus Pritchardinychus Wainstein is proposed as a synonym of the subgenus Reckiella. Moreover, based on female morphological characters, four species groups (*coffeae*, *exsiccator*, *iseilemae*, and *peruvianus*) and 11 species subgroups (*aceris*, *biharensis*, *coffeae*, *comptus*, *exsiccator*, *gossypii*, *iseilemae*, *peruvianus*, *pritchardi*, *smithi*, and *subnudus*) are suggested in the subgenera *Oligonychus* and *Reckiella*. Fourteen *Oligonychus* species are proposed as species inquirendae, and potential cryptic species complexes in the genus *Oligonychus* are briefly highlighted. It is agreed that the clunal seta *h_1_* is always absent, while the para-anal setae *h_2_* and *h_3_* are always present in the genus *Oligonychus.* A key to subgenera, species groups, and species subgroups of the genus *Oligonychus* is provided.

## ﻿Introduction

*Oligonychus* Berlese (Acari: Prostigmata: Tetranychidae) is the largest genus of the spider mites, comprising > 200 species, and its members have been reported throughout the world (Migeon and Dorkeld 2021). A range of feeding specificity occurs throughout the genus, with polyphagous, oligophagous and monophagous species present on both broad and narrow leaved commercial (fruits, agronomic crops, etc.) and non-commercial (wild trees, shrubs, grasses, etc.) host plants (Pritchard and Baker 1955; Jeppson et al. 1975; Beard et al. 2003; Matsuda et al. 2012; Migeon and Dorkeld 2021). Some economically significant species, e.g., the date palm mite *O.afrasiaticus* (McGregor), the tea red spider mite *O.coffeae* (Neitner), the banks grass mite/new world date mite *O.pratensis* (Banks), and the avocado brown mite *O.punicae* (Hirst), have been spread across the world and are now widely distributed (Jeppson et al. 1975; Migeon and Dorkeld 2021).

The authenticity of sub-generic division of the genus *Oligonychus* (Wainstein 1960; Tuttle and Baker 1968) always remains questionable, due to the use of inconsistent characters, e.g., striae pattern on dorsal opisthosoma and number of tactile setae on tibia I (Meyer 1974, 1987; Bolland et al. 1998; Beard et al. 2003, 2008; Khanjani et al. 2018; Li et al. 2018, 2019). So, to confirm the current taxonomic status of the six subgenera of *Oligonychus* suggested by Tuttle and Baker (1968), a comprehensive morphological investigation based on all known *Oligonychus* species is needed.

The species identity in the genus *Oligonychus* is usually challenging due to the limited number of potential diagnostic characters, presence of intraspecific variation, minute differences in male aedeagus morphology and interspecific similarities in females (Pritchard and Baker 1955; Meyer 1974, 1987; Jeppson et al. 1975; Li et al. 2018). Mostly, *Oligonychus* species have been differentiated based only on the aedeagus morphology (Pritchard and Baker 1955; Meyer 1974, 1987). In addition, specimens of both sexes are frequently required for precise identification of *Oligonychus* species (Ben-David 2008; Meyer 1987). The aedeagal traits could be unreliable and confusing when male specimens were not mounted in a precisely lateral position, and in some cases the aedeagus was neither described nor illustrated (e.g., *O.mangiferus* Rahman & Sapra, 1940). Also, intraspecific variations in aedeagus shape or variation in the interpretation of aedeagus shape that can manifest as intraspecific variation, have been observed in species described from various geographical localities (e.g., *O.pratensis*; McGregor 1939; Pritchard and Baker 1955; Meyer 1959, 1974, 1987; Baker and Pritchard 1962; Estebanes and Baker 1968; Jeppson et al. 1975; Tuttle et al. 1976; Baker and Tuttle 1994). Furthermore, aedeagus shape may appear variable at different levels of focus under the microscope (e.g., *O.ephamnus* Beard & Walter, 2003). There are 17 *Oligonychus* species that have been described based only on females with the males remaining unknown, e.g., *O.mactus* Tseng and *O.nielseni* Reeves (Reeves 1963; Tseng 1990), and a few species that were inadequately described, e.g., *O.kobachidzei* (Reck) and *O.stenoperitrematus* (Ugarov and Nikolskii), with important morphological information of male/female not included (Ugarov and Nikolskii 1937; Reck 1947). Some species, e.g., *O.caucasicus* (Reck) and *O.daleae* Tuttle, Baker & Abbatiello, were reported as new to science, without making any remarks regarding related or similar species (Reck 1956; Tuttle et al. 1976). This lack in clarity has resulted in the suggested existence of several species complexes within the genus *Oligonychus* e.g., *coffeae* complex and *pratensis* complex (Pritchard and Baker 1955; Meyer 1987; Ehara and Gotoh 2007; Lara et al. 2017; Khanjani et al. 2018; Li et al. 2018). Consequently, there is a great need for developing an integrative taxonomic approach to clarify the actual status of many closely related *Oligonychus* species and for establishing truly diagnostic characters for accurate and consistent species separation.

The presence of two pairs of para-anal setae (*h_2_* and *h_3_*) is one of the important distinguishing character of *Oligonychus* (Lindquist 1985; Beard et al. 2003, 2008; Seeman and Beard 2011; Arabuli and Gotoh 2018; Khanjani et al. 2018; Li et al. 2019). However, there are contradictions found among different taxonomists even when it comes to identifying the *h_2_* setae in *Oligonychus* species (Pritchard and Baker 1952, 1955; Meyer 1974, 1987; Jeppson et al. 1975; Lindquist 1985; Bolland et al. 1998; Knihinicki and Flechtmann 1999; Flechtmann and Etienne 2006; Beard et al. 2008; Arabuli and Gotoh 2018; Khanjani et al. 2018; Li et al. 2019).

Keeping in view the importance of taxonomic adversities in the genus *Oligoncyhus*, the objectives of the present study were to i) assess the current taxonomic status of the sub-generic division of *Oligonychus*, ii) classify all species of *Oligonychus* into species groups and subgroups based on consistent morphological characters, iii) construct a diagnostic key to subgenera, groups and subgroups of *Oligonychus*, and iv) highlight or discuss the doubtful species, species complexes and contradiction/confusion in the identification of para-anal setae in *Oligonychus*.

## ﻿Materials and methods

The taxonomic literature of 211 *Oligonychus* species was critically reviewed to confirm the current status of subgeneric division and doubtful and closely related *Oligonychus* species, create species groups, and prepare a key for their identification; in addition to discussing the confusion/contradiction associated with the naming of para-anal setae. To verify the consistency in expression of some female morphological characters and their significance in creating species groups and subgroups within the genus *Oligonychus*, numerous spider mite samples were collected and observed from Egypt, Mexico, Pakistan, Saudi Arabia, USA, and Yemen. In addition, mite samples of some other closely or distantly related Tetranychini genera viz. *Tetranychus* Dufour, *Eotetranychus* Oudemans, *Mixonychus* Meyer & Ryke, *Neotetranychus* Trägårdh, *Sonotetranychus* Tuttle et al., and *Schizotetranychus* Trägårdh, were collected from various localities in different regions of Saudi Arabia, to confirm the absence/presence and shape and position of the clunal (*h_1_*) and para-anal setae (*h_2_* and *h_3_*). The nomenclature of Grandjean (1939, 1944a, 1944b, 1947) was followed for body setae, and Lindquist (1985) for leg chaetotaxy and other terminologies.

## ﻿Results and discussion

### Family Tetranychidae Donnadieu

**Subfamily Tetranychinae Berlese**

### Tribe Tetranychini Reck

#### ﻿Genus Oligonychus

Taxon classificationAnimaliaTrombidiformesTetranychidae

Berlese, 1886.

0F74BC4B-31B2-544D-9316-F17DB69A0F93

Oligonychus Berlese, 1886: 24, Pritchard and Baker 1955: 270, Wainstein 1960: 203, Tuttle and Baker 1968: 116, Meyer 1974: 248, Mitrofanov 1977: 1801–1802, Meyer 1987: 142, Beard et al. 2003: 51–78, Khanjani et al. 2018: 223–287, Li et al. 2019: 1071–1106.

##### Type species.

*Heteronychusbrevipodus* Targioni-Tozzetti, 1878: 255.

##### Diagnosis.

(Based on: Pritchard and Baker 1955; Tuttle and Baker 1968; Meyer 1974, 1987; Beard et al. 2003, 2008; Khanjani et al. 2018). Empodia well developed, claw-like with proximoventral hairs (except male leg I with hairs modified into spur) that are as long as or shorter than empodial claw on most of legs; two pairs of duplex setae on tarsus I, distal and adjacent; opisthosoma with 11 pairs of dorsal setae (*c_1-3_*, *d_1-2_*, *e_1-2_*, *f_1-2_*, *h_2-3_*; n.b. setae *h_2_* and *h_3_* usually inserted ventrally); clunal setae *h_1_* always absent.

###### ﻿Taxonomic review of the genus *Oligonychus*

The genus *Oligonychus* was erected by Berlese (1886), based on *Heteronychusbrevipodus* Targioni-Tozzetti as the type species (specimen was a nymph), reported from the Holly Oak (*Quercusilex* L., Fagaceae) in Italy (Targioni-Tozzetti 1878). Because Berlese (1886) did not clearly describe the presence of proximoventral hairs on the empodial claws in the diagnosis of *Oligonychus*, Zacher (1913) created a new genus, *Paratetranychus*, and described the presence of proximoventral hairs in it. McGregor (1950) followed the work of Zacher (1913) by giving priority to *Paratetranychus* over *Oligonychus*, and placed all *Oligonychus* species within *Paratetranychus*. Five years later, Pritchard and Baker (1955) redefined the genus *Oligonychus*, and synonymized the genera *Paratetranychus* and *Tacebia* (Yokoyama 1929) with *Oligonychus.*

### Subdivision of the genus *Oligonychus*

The genus *Oligonychus* has a history of subdivision into species groups (Pritchard and Baker 1955; Ehara 1999) and subgenera (Wainstein 1960; Tuttle and Baker 1968). Initially, Pritchard and Baker (1955) divided *Oligonychus* species into five species groups viz. *ununguis*, *peruvianus*, *pritchardi*, *pratensis*, and *mcgregori*. The *ununguis* group was further divided into five species subgroups viz. *aceris*, *bicolor*, *boudreauxi*, *subnudus*, and *ununguis*. This grouping was based on both male and female morphological characters, including the shape or orientation of the male aedeagus, number of tactile setae on tibia I, number of tactile setae on tarsus I proximal to the proximal duplex setae and ventrally near or beyond the duplex setae, number of proximoventral hairs on the empodium, the pattern of striation on the female dorsal opisthosoma, shape of dorsal body setae, and the shape of the peritreme (Pritchard and Baker 1955). Another species group, the *clavatus* species group, was subsequently created by Ehara (1999) based on the number of tactile setae on tibia I, aedeagal morphology and female body color, and included *O.clavatus* (Ehara) and *O.pustulosus* Ehara.

Wainstein (1960) proposed five sub-genera of *Oligonychus*, namely *Oligonychus* Berlese, *Homonychus* Wainstein, *Metatetranychoides* Wainstein, *Pritchardinychus* Wainstein, and *Paratetranychus* Zacher. This sub-generic division was based on both male and female morphological characters, including the length of dorsal body setae, dorsal setae set on tubercles or not, the total number of setae on each of female tibia I, tibia II and tarsus I, and the morphology of the male aedeagus. Three of these sub-genera were further categorized into species groups based on the chaetotaxy of legs I and II. The subgenus Oligonychus was divided into three species groups: *boudreauxellus*, *bakerellus*, and *berlesellus*; the subgenus Paratetranychus was divided into two groups: *ununguellus* and *zacherellus*; and the subgenus Pritchardinychus was divided into three groups: *pritchardellus*, *pratensellus*, and *mcgregorellus*. Later, Tuttle and Baker (1968) synonymized the subgenus Paratetranychus with the subgenus Oligonychus, and retained four of the sub-genera created by Wainstein (1960). Tuttle and Baker (1968) then created two more sub-genera, namely *Wainsteiniella* Tuttle & Baker and *Reckiella* Tuttle & Baker. This sub-generic division was again based on both male and female characters, including the pattern of the striation of the female dorsal opisthosoma, shape and orientation of male aedeagus, length of dorsal body setae, and total number of tactile setae on tibia I. Tuttle and Baker (1968) relied heavily on one of the female morphological characters to differentiate these six subgenera, the pattern of the striae on the dorsal opisthosoma. Mitrofanov (1977) also used this character to raise the sub-genera *Metatetranychoides*, *Pritchardinychus*, and *Homonychus* to genus level, and to erect a new genus, *Neonychus*, with *O.licinus* Baker & Pritchard as type species. Meyer (1987) found that the pattern of dorsal striae on the female opisthosoma to be a variable character, and disagreed with the subgeneric divisions of *Oligonychus* made by Wainstein (1960) and Tuttle and Baker (1968). The sub-genera are not always well distinguished and several morphological characters, such as the pattern of striae between dorsal setal pairs *e_1_* and *f_1_* on the female and the number of tactile setae on tibia I, have been found to be variable (Meyer 1987). Such variable characters cannot be used to reliably separate the subgenera of *Oligonychus* (Meyer 1987; Beard et al. 2003, 2008; Li et al. 2018). These sub-divisions were made for practical rather than phylogenetic reasons, and have resulted in a somewhat artificial classification (Helle et al. 1981; Meyer 1987).

Bolland et al. (1998) supported homogeneity within only the two sub-genera viz. *Reckiella* and *Oligonychus*, based on biological, morphological, and molecular data, but felt that the homogeneity of the remaining four sub-genera (*Homonychus*, *Metatetranychoides*, *Pritchardinychus*, and *Wainsteiniella*) requires further investigation to confirm their validity. Additionally, molecular investigations have revealed the presence of “polyphyly” in the genus *Oligonychus* (Navajas et al. 1996; Ben-David et al. 2007; Matsuda et al. 2014).

### Species complexes in the genus *Oligonychus*

Several species complexes within the genus *Oligonychus* have been suggested by various authors in the past, for example a coffeae complex, *pratensis* complex, *perseae* complex, *subnudus* complex, *sacchari* complex, and *ununguis* complex (Pritchard and Baker 1955; Cromroy 1958; Meyer 1987; Ehara and Gotoh 2007; Lara et al. 2017; Li et al. 2018), and these are difficult to resolve based on morphology (Pritchard and Baker 1955). The *coffeae* complex sensu Ehara & Gotoh comprises four morphologically similar species, namely *O.coffeae*, *O.gotohi* Ehara, *O.castaneae* Ehara & Gotoh, and *O.amiensis* Ehara & Gotoh (Ehara and Gotoh 2007). Originally, the Japanese population of *O.gotohi* was considered to represent a single species, however, genetic crossing studies revealed that this population is a complex of three reproductively isolated species (*O.gotohi*, *O.castaneae*, and *O.amiensis*) (Ehara and Gotoh 2007; Gotoh et al. 2007). Moreover, some variation in morphological characters, e.g., aedeagus shape and number of tactile setae on tarsus II, have been observed in various taxa identified as *O.coffeae* reported from different geographical localities (Pritchard and Baker 1955; Baker and Pritchard 1960; Ehara 1969, 1999; Meyer 1974, 1987; Wang 1981), highlighting the possibility that further cryptic species could be separated within this complex (Ehara and Gotoh 2007).

The *pratensis* complex sensu Pritchard & Baker has been recognized by various authors based on observed variations or differences in some morphological characters among different populations identified as *O.pratensis*, e.g., aedeagus shape and striae pattern on dorsal hysterosoma (Pritchard and Baker 1955; Meyer 1974; Li et al. 2018). A subnudus complex was suggested by Pritchard and Baker (1955), when morphological variations in shape/length of some hysterosomal setae and the stylophore were observed between two populations of *O.subnudus* (McGregor) from two different localities in the United States.

Based on the variations in descriptions and illustrations of two morphologically similar *Oligonychus* species, *O.sacchari* (McGregor) and *O.saccharinus* Baker & Pritchard (McGregor 1950; Pritchard and Baker 1955; Baker and Pritchard 1960; Meyer 1974), the *sacchari* complex was proposed by Khanjani et al. (2018). Although, Meyer (1974) comprehensively discussed the morphological differences between these two closely related species, their taxonomic identities remain doubtful, and require further investigations through the combined use of morphological and molecular data (Khanjani et al. 2018).

The possibility of an *ununguis* complex was suggested by Pritchard and Baker (1955) to include *O.coniferarum* (McGregor), *O.mangiferus*, *O.peronis* Pritchard & Baker, *O.punicae*, and *O.ununguis* (Jacobi). The taxonomic identities of most species in the *ununguis* complex remain questionable, and the females are indistinguishable. The minute differences in the shape of the aedeagus and the size of female palp spinneret are often used for differentiating these closely related species (Pritchard and Baker 1955; Meyer 1987; Khanjani et al. 2018). However, some of these species e.g., *O.mangiferus*, *O.punicae*, and *O.vitis*, are very close morphologically, can be exceedingly difficult to differentiate as separate species, and are part of the “greatest taxonomic problem” in the genus *Oligonychus* (Meyer 1987; Khanjani et al. 2018).

### Para-anal setae in the genus *Oligonychus*

In many genera of the tribe Tetranychini Reck, three pairs of *h* setae (*h_1_*, *h_2_*, and *h_3_*) are consistently present on the fifth segment (H) of opisthosoma (Pritchard and Baker 1955; Lindquist 1985; Bolland et al. 1998). However, one of these setae (*h_1_* or the clunals) is absent in some Tetranychini genera, e.g., *Oligonychus* and *Tetranychus* (Pritchard and Baker 1955; Lindquist 1985; Bolland et al. 1998; Seeman and Beard 2011; Alatawi and Kamran 2018; Khanjani et al. 2018). Earlier, Pritchard and Baker (1955) believed that seta *h_1_* (clunal seta) is consistently present and *h_2_* (one of a pair of para-anal setae displaced terminally to become a post anal seta) is absent in *Oligonychus*. Later, Lindquist (1985) analyzed and discussed the relative position and shape of the para-anal setae (*h_2_* and *h_3_*) with respect to setal homologies and concluded that the clunal seta *h_1_* is apparently absent in *Oligonychus*, whereas seta *h_2_* is consistently present (Lindquist 1985), as previously explained by many authors (Oudemans 1930; Pritchard and Baker 1952; Attiah 1970). According to Lindquist (1985) and Seeman and Beard (2011), para-anal setae *h_2_* and *h_3_*, are consistently present in *Oligonychus*. Confusion is generated when simple positions are used to name setae rather than homologies. Lindquist (1985) and Seeman and Beard (2011) use homologies and state that “two pairs of para-anal setae *h_2_* and *h_3_*, are consistently present in *Oligonychus* and related genera”. Many authors have followed the work of Lindquist (1985), and also consider setae *h_1_* to be absent, and *h_2_* and *h_3_* to be present in *Oligonychus* (Beard et al. 2003, 2008; Kamayev 2017; Li et al. 2017, 2018, 2019; Arabuli and Gotoh 2018; Khanjani et al. 2018). Pritchard and Baker (1952) originally assumed that seta *h_1_* (clunal) was present or absent, with two pairs of para-anal setae present; however, Prtichard and Baker (1955) altered this view and assumed the clunal setae to always be present. They stated that there are two pairs of para-anal setae, and that the posterior pair get displaced terminally to become a post-anal seta, and that it is this seta that is absent in two genera (*Oligonychus* and *Tetranychus*). Meyer (1987) and Bolland et al. (1998) also use a positional approach to naming setae and assume the clunals to be consistently present, but interpret setae *h_2_* or *h_3_* as absent in *Oligonychus* and *Tetranychus*. Thus the setae are named as one pair of clunals and two pair of para-anals, and as a consequence, the statement “one pair of para-anal setae is present in *Oligonychus* and related genera” appears to be contradictory to what other authors believe. Many authors also mention the presence of *h_1_* and only one pair of para-anal setae (either *h_2_* or *h*_3_) in the genus diagnosis and descriptions/illustrations of different *Oligonychus* species (Rimando 1962; Tuttle and Baker 1968; Chaudhri et al. 1974; Meyer 1974; Jeppson et al. 1975; Tuttle et al. 1976; Zaher et al. 1982; Tseng 1990; Gupta and Gupta 1994; Smiley and Baker 1995; Ehara 1999; Knihinicki and Flechtmann 1999; Flechtmann and Etienne 2006; Ehara and Gotoh 2007; Zeity 2015, 2016; Alatawi and Kamran 2018).

#### ﻿Subgeneric division of the genus *Oligonychus*

### Subgenus Oligonychus Berlese

**Type species**. *Heteronychusbrevipodus* Targioni-Tozzetti, 1878: 255.

**Diagnosis (based on male)**. Male aedeagus with shaft bending ventrad, downturned part mostly tapering distally, forming an acute or blunt tip.

### Subgenus Reckiella Tuttle & Baker

**Type species**. *Tetranychuspratensis* Banks, 1912: 97.

**Diagnosis (based on male).** Male aedeagus with shaft bending dorsad, or shaft initially bends dorsad then distal part turned ventrad, upturned part usually without tapering end, distally forming knob, sigmoidal shape and blunt or rounded tip.

Only two subgenera are hereby recognized: *Oligonychus* Berlese and *Reckiella* Tuttle & Baker, instead of five and six subgenera as proposed by Wainstein (1960) and Tuttle and Baker (1968), respectively. The subgenera *Homonychus*, *Metatetranychoides* and Wainsteiniella are considered to be synonyms of the subgenus Oligonychus, and subgenus Pritchardinychus is recommended as a synonym of the subgenus Reckiella. In total, 76 species are placed in the subgenus Oligonychus, whereas 118 Oligonychus species are designated to the subgenus Reckiella. However, approximately 17 *Oligonychus* species could not be assigned to any of the two subgenera, because their descriptions were based only on females, with males remaining unknown in the original and subsequent descriptions.

In the present study, we suggest using the male aedeagus shape and its orientation as a consistent and strong morphological character to redefine the two valid *Oligonychus* subgenera, instead of using inconsistent or variable characters, e.g., striation pattern on dorsal hysterosoma and number of tactile setae on tibia I (Wainstein 1960; Tuttle and Baker 1968; Meyer 1987). The proposed suggestion also agreed well with the molecular separation of various *Oligonychus* species into two groups, which successfully coincided with their morphological grouping based on male aedeagus i.e., aedeagus upturned vs. aedeagus downturned (Ben-David et al. 2007; Matsuda et al. 2012; Unpublished results). This study also supported the findings of Meyer (1974, 1987), who was the first to disagree with the subgeneric divisions of *Oligonychus* (Wainstein 1960; Tuttle and Baker 1968), due to the inconsistency of diagnostic characters, e.g., striation pattern on dorsal opisthosoma and number of tactile setae on tibia I. Meyer (1987) found that some of the African *Oligonychus* species, e.g., *O.andrei* Gutierrez and *O.pennisetum*, would not fit in any of the six *Oligonychus* subgenera (Tuttle and Baker 1968). Subsequently, more authors disagreed with the six-subgeneric *Oligonychus* system (Flechtmann and Alves 1976; Helle et al. 1981; Bolland et al. 1998; Beard et al. 2003, 2008; Khanjani et al. 2018; Li et al. 2018). However, there are still authors who continue using the *Oligonychus* six-subgeneric system (Kamayev 2017; Li et al. 2019), without confirming the validity of characters that were initially devised to erect these subgenera (Tuttle and Baker 1968).

Based on the upturned aedeagus and tibia I with nine tactile setae (Tuttle and Baker 1968; Jeppson et al. 1975), we here synonymize the subgenus Pritchardinychus with the subgenus Reckiella. Both of these subgenera were previously separated using an inconsistent character of regarding the dorsal hysterosomal striae on the female (Meyer 1987) – longitudinal striae only between setae *f_1_-f_1_* in *Reckiella* or transverse striae on entire hysterosoma in *Pritchardinychus* (Tuttle and Baker 1968). In the present study, we observed longitudinal, irregular longitudinal or oblique striae present between both *e_1_-e_1_* and *f_1_-f_1_* setae in 10 Oligonychus species of the valid subgenus Reckiella, and longitudinal striae between only *e_1_-e_1_* setae in a species (*O.andrei* Gutierrez) of *Reckiella* (Zacher 1921; Baker and Pritchard 1960; Rimando 1962; Meyer 1964, 1965, 1974, 1987; Ehara 1966; Gutierrez 1966, 1967; Lo and Ho 1989; Tseng 1990). Furthermore, 13 Oligonychus species of the valid subgenus Reckiella with upturned aedeagus have seven or less than seven tactile setae on tibia I rather than nine setae. Also, the pattern of opisthosomal/hysterosomal striae of some of these 13 species varies, e.g., reticulated pattern of irregular elongate elements in the case of *O.comptus* Meyer & Bolland, whole hysterosoma with transverse striae in the case of *O.anonae* Paschoal, *O.beeri* Estebanes & Baker, *O.chiapensis* Estebanes & Baker and *O.iseilemae* (Hirst), transverse except V-shaped/irregular pattern between *e_1_-e_1_* setae in *O.megandrosoma* Flechtmann & Alves, and transverse except slightly U-shaped pattern between *e_1_-f_1_* area in *O.poutericola* Feres & Flechtmann (Hirst 1924; Baker and Pritchard 1962; Estebanes and Baker 1968; Livshits 1968; Gutierrez 1969; Paschoal 1970; Meyer 1974, 1987; Flechtmann and Alves 1976; Meyer and Bolland 1984; Feres and Flechtmann 1986; Mendonca et al. 2010).

We also synonymized the subgenera that have a downturned aedeagus, i.e., *Homonychus*, *Metatetranychoides*, and *Wainsteiniella* (Tuttle and Baker 1968; Jeppson et al. 1975), with the subgenus Oligonychus. Previously, these four subgenera were also diagnosed and separated based mainly on the inconsistent character of dorsal hysterosomal striae of the female (Meyer 1987), with *Oligonychus* and *Wainsteiniella* having entirely transverse striae, and *Homonychus* with longitudinal and *Metatetranychoides* with irregular striae between only setae *e_1_-e_1_* (Tuttle and Baker 1968; Jeppson et al. 1975). Of the four subgenera, *Wainsteiniella* was further diagnosed with shorter dorsal body setae (Tuttle and Baker 1968). However, we observed that some Oligonychus (Oligonychus) species, or populations of a species, have short dorsal setae with V-shaped, longitudinal, irregular, or oblique striae between *d_1_-e_1_* setal area instead of entirely transverse, e.g., *O.hondoensis* (Ehara)) and *O.plumosus* Estebanes & Baker (Pritchard and Baker 1955; Tuttle et al. 1976). In addition, the subgenus Oligonychus was also differentiated by having seven tactile setae on tibia I (Tuttle and Baker 1968), and we found some species with more than seven, e.g., *O.bambusae* Karuppuchamy & Mohanasundaram and *O.smithi* Cromroy, or fewer than than seven, e.g., *O.alpinus* (McGregor). Moreover, *O.bambusae* possesses longitudinal striae between setae *f_1_-f_1_* (McGregor 1936; Cromroy 1958; Karuppuchamy and Mohanasundaram 1988; Baker and Tuttle 1994).

#### ﻿Subdivision of *Oligonychus* species into groups and subgroups

In the present study, four species groups and 11 species subgroups are recognized under the valid subgenera of *Oligonychus* and *Reckiella*, based on the combination of three morphological characters of the adult female: the number of tactile setae on tibiae I and II, the length of dorsal hysterosomal setae *c_1_*, and the pattern of striae on the dorsal hysterosoma. These characters were previously used to erect species groups by Pritchard and Baker (1955) and subgenera by Tuttle and Baker (1968) in *Oligonychus*.

#### ﻿Species groups and subgroups in the subgenus Oligonychus

The Oligonychus (Oligonychus) is subdivided into two species groups, the *peruvianus* species group (Pritchard and Baker 1955) and the newly proposed *coffeae* species group. The *peruvianus* species group is further categorized into two newly proposed species subgroups, the *smithi* species subgroup and the *peruvianus* species subgroup; whereas the *coffeae* species group is categorized into three species subgroups, *subnudus* species subgroup (Pritchard and Baker 1955), *aceris* species subgroup (Pritchard and Baker 1955), and the newly proposed *coffeae* species subgroup.

The following four species of the subgenus Oligonychus could not be assigned to any species group/subgroup, because they were briefly described and certain key characters of the female were not included:

*O.brevipilosus* (Zacher, 1932)

*O.kobachidzei* (Reck, 1947)

*O.meifengensis* Lo & Ho, 1989

*O.nuptialis* (Zacher, 1932)

### *peruvianus* species group (sensu Pritchard & Baker, 1955)

**Exemplar species.***Tetranychusperuvianus* McGregor, 1917: 581.

**Diagnosis (based on female).** More than seven (eight or nine) tactile setae on tibia I.

### *smithi* new species subgroup

**Exemplar species.***Oligonychussmithi* Cromroy, 1958: 61.

**Diagnosis (based on female).** More than seven (eight or nine) tactile setae on tibia I, and dorsal hysterosomal setae *c_1_* long, reaching well beyond bases of setae *d_1_.* This subgroup comprises two species:

*O.bambusae* Karuppuchamy & Mohanasundaram, 1988

*O.smithi* Cromroy, 1958

### *peruvianus* new species subgroup

**Exemplar species.***Tetranychusperuvianus* McGregor, 1917: 581.

**Diagnosis (based on female).** More than seven (eight or nine) tactile setae on tibia I, and dorsal hysterosomal setae *c_1_* short (almost one-half to three-fourths as long as the distance between *c_1_-d_1_*), not reaching bases of setae *d_1_.* This subgroup includes three species:

*O.peruvianus* (McGregor, 1917)

*O.perseae* Tuttle, Baker & Abbatiello, 1976

*O.sumatranus* Ehara, 2004

### *coffeae* new species group

**Exemplar species.***Acaruscoffeae* Nietner, 1861: 31.

**Diagnosis (based on female).** Seven or less than seven (five or six) tactile setae on tibia I.

### *subnudus* species subgroup (sensu Pritchard and Baker 1955)

**Exemplar species.***Paratetranychussubnudus* McGregor, 1950: 354

**Diagnosis (based on female).** Seven or less than seven (five or six) tactile setae on tibia I, and dorsal hysterosomal setae *c_1_* short (almost one half to three-fourths as long as the distance between *c_1_*-*d_1_*), not reaching bases of setae *d_1_.* This subgroup includes 18 species:

*O.baipisongis* Ma & Yuan, 1976

*O.boudreauxi* Pritchard & Baker, 1955

*O.clavatus* (Ehara, 1959)

*O.cunliffei* Pritchard & Baker, 1955

*O.hondoensis* (Ehara, 1954)

*O.karamatus* (Ehara, 1956)

*O.livschitzi* Mitrofanov & Bossenko, 1975

*O.laricis* Reeves, 1963

*O.milleri* (McGregor, 1950)

*O.pinaceus* Mitrofanov & Bossenko, 1975

*O.pini* Tuttle, Baker & Abbatiello, 1976

*O.pityinus* Pritchard & Baker, 1955

*O.plumosus* Estebanes & Baker, 1968

*O.subnudus* (McGregor, 1950)

*O.tuberculatus* Estebanes & Baker, 1968

*O.verduzcoi* Estebanes & Baker, 1968*

*O.yasumatsui* Ehara & Wongsiri, 1975

*O.yuae* Tseng, 1975

### *aceris* species subgroup (sensu Pritchard and Baker 1955)

**Exemplar species.***Acarusaceris* Shimer, 1869: 320

**Diagnosis (based on female).** Five or six tactile setae on tibia I, and dorsal hysterosomal setae *c_1_* long, reaching to (sub-equal to the distance between *c_1_-d_1_*) or well beyond bases of setae *d_1_.* This subgroup comprises of five species:

*O.aceris* (Shimer, 1869)

*O.alpinus* (McGregor, 1936)

*O.endytus* Pritchard & Baker, 1955

*O.gambelii* Tuttle & Baker, 1968

*O.pustulosus* Ehara, 1962

### *coffeae* new species subgroup

**Exemplar species.***Acaruscoffeae* Nietner, 1861: 31.

**Diagnosis (based on female).** Seven tactile setae on tibia I, and dorsal hysterosomal setae *c_1_* long, reaching to (sub-equal to the distance between *c_1_-d_1_*) or well beyond bases of setae *d_1_.* It comprises of 44 species:

*O.bicolor* (Banks, 1894)

*O.brevipodus* (Targioni-Tozzetti, 1878)

*O.buschi* (Reck, 1956)

*O.chamaecyparisae* Ma & Yuan, 1976

*O.camelliae* Ehara & Gotoh, 2007

*O.castaneae* Ehara & Gotoh, 2007

*O.coffeae* (Nietner, 1861)

*O.coniferarum* (McGregor, 1950)

*O.cubensis* (Livshits, 1968)

*O.gotohi* Ehara, 1999

*O.gutierrezi* Parsi, 1979

*O.hamedaniensis* Khanjani, Khanjani & Seeman, 2018

*O.ilicis* (McGregor, 1917)

*O.judithae* Meyer, 1974

*O.juniperi* Tuttle, Baker & Abbatiello, 1976

*O.lagodechii* Livshits & Mitrofanov, 1969

*O.longiclavatus* (Reck, 1953)

*O.letchworthi* Reeves, 1963

*O.mangiferus* (Rahman & Sapra, 1940)

*O.metasequoiae* Kuang, 1992

*O.mitis* Beglyarov & Mitrofanov, 1973

*O.neocastaneae* Arabuli & Gotoh, 2018

*O.newcomeri* (McGregor, 1950)

*O.ochoai* Meyer & Vargas, 1999

*O.pruni* Mitrofanov & Zapletina, 1973

*O.penai* Rimando, 1962

*O.perditus* Pritchard & Baker, 1955

*O.peronis* Pritchard & Baker, 1955

*O.piceae* (Reck, 1953)

*O.platani* (McGregor, 1950)

*O.ponmanaiensis* Karuppuchamy & Mohanasundaram, 1987

*O.punicae* (Hirst, 1926)

*O.qilianensis* Ma & Yuan, 1982

*O.santoantoniensis* Feres & Flechtmann, 1995

*O.shojaeii* Khanjani, Khanjani & Seeman, 2018

*O.steinhaueri* Flechtmann & Baker, 1970

*O.tshimkenticus* Wainstein, 1956

*O.tsudomei* Ehara, 1966

*O.ununguis* (Jacobi, 1905)

*O.viranoplos* Flechtmann, 1993

*O.viridis* (Banks, 1894)

*O.vitis* Zaher & Shehata, 1965

*O.yothersi* (McGregor, 1914)

*O.yusti* McGregor, 1959

#### ﻿Species groups and subgroups in the subgenus Reckiella

Oligonychus (Reckiella) is subdivided into two new species groups, the *iseilemae* species group and the *exsiccator* species group. The *iseilemae* species group is further categorized into two new species subgroups, the *comptus* species subgroup and the *iseilemae* species subgroup; whereas the *exsiccator* species group is categorized into four new species subgroups: the *pritchardi* species subgroup, the *biharensis* species subgroup, the *gossypii* species subgroup, and the *exsiccator* species subgroup.

The following two species of the subgenus Reckiella could not be assigned to any species group/subgroup, because they were briefly described and/or certain key characters of the female were not included:

*O.annonicus* (McGregor, 1955)

*O.stenoperitrematus* (Ugarov & Nikolskii, 1937)

### *iseilemae* new species group

**Exemplar species.***Paratetranychusiseilemae* Hirst, 1924: 524.

**Diagnosis (based on female).** Seven or less than seven (five or six) tactile setae on tibia I.

### *comptus* new species subgroup

**Exemplar species.***Oligonychuscomptus* Meyer & Bolland, 1984: 218.

**Diagnosis (based on female).** Seven or less than seven (five or six) tactile setae on tibia I, and dorsal hysterosoma with a reticulate pattern of irregular and elongate elements medially. This subgroup includes only one species:

*O.comptus* Meyer & Bolland, 1984

### *iseilemae* new species subgroup

**Exemplar species.***Paratetranychusiseilemae* Hirst, 1924: 524.

**Diagnosis (based on female).** Seven or less than seven (five or six) tactile setae on tibia I, and dorsal hysterosoma ornamented with simple striations medially, reticulate pattern absent. This subgroup comprises of 12 species:

*O.acugni* (Livshits, 1968)

*O.amnicolus* Meyer, 1974

*O.anonae* Paschoal, 1970

*O.bagdasariani* Baker & Pritchard, 1962

*O.beeri* Estebanes & Baker, 1968

*O.chiapensis* Estebanes & Baker, 1968

*O.fileno* Mendonca, Navia & Flechtmann, 2010

*O.iseilemae* (Hirst, 1924)

*O.megandrosoma* Flechtmann & Alves, 1976

*O.occidentalis* Gutierrez, 1969

*O.poutericola* Feres & Flechtmann, 1986

*O.themedae* Meyer, 1974

### *exsiccator* new species group

**Exemplar species.***Tetranychusexsiccator* Zehntner, 1897: 572.

**Diagnosis (based on female).** More than seven (eight, nine or rarely ten) tactile setae on tibia I.

Due to unavailability of morphological information about the pattern of dorsal hysterosomal striae in female, the following one species could not be assigned to any of subgroup of the species group *exsiccator*:

*O.bruneri* (Livshits, 1968)

### *pritchardi* new species subgroup

**Exemplar species.***Paratetranychuspritchardi* McGregor, 1950: 350.

**Diagnosis (based on female).** More than seven (eight, nine or rarely ten) tactile setae on tibia I, five or six tactile setae on tibia II, and dorsal hysterosoma with uniform or wavy transverse striae between setae *d_1_-f_2_* area, rarely with a mixture of wavy and oblique striae medially posterior to setae *f_1_*. This subgroup comprises of 13 species:

*O.calcis* Baker & Pritchard, 1960

*O.festucolus* Beard & Walter, 2003

*O.flechtmanni* Tuttle, Baker & Sales, 1977

*O.longipenis* Feres & Flechtmann, 1995

*O.mimosae* Baker & Pritchard, 1962

*O.psidii* Flechtmann, 1967

*O.psidium* Estebanes & Baker, 1968

*O.pritchardi* (McGregor, 1950)

*O.propetes* Pritchard & Baker, 1955

*O.quasipropetes* Flechtmann, 1981

*O.quercus* Tuttle, Baker & Abbatiello, 1976

*O.tiwakae* Gutierrez, 1978

*O.veranerae* Baker & Pritchard, 1962

### *biharensis* new species subgroup

**Exemplar species.***Paratetranychusbiharensis* Hirst, 1924: 69.

**Diagnosis (based on female).** More than seven (eight, nine or rarely ten) tactile setae on tibia I, seven tactile setae on tibia II, and dorsal hysterosoma with uniform or wavy transverse striae between setae *d_1_-f_2_* area rarely with a mixture of wavy and oblique striae medially posterior to setae *f_1_*. This subgroup includes ten species:

*O.antherus* Rimando, 1962

*O.apohadrus* Meyer, 1987

*O.biharensis* (Hirst, 1924)

*O.hadrus* Pritchard & Baker, 1955

*O.hova* Gutierrez, 1966

*O.imberbei* Meyer, 1974

*O.macrostachyus* Baker & Tuttle, 1972

*O.malawiensis* Meyer, 1974

*O.pemphisi* Gutierrez, 1970

*O.sapienticolus* Gupta, 1976

### *gossypii* new species subgroup

**Exemplar species.***Paratetranychusgossypii* Zacher, 1921: 183.

**Diagnosis (based on female).** More than seven (eight, nine or rarely ten) tactile setae on tibia I, and dorsal hysterosoma with various patterns of striae: longitudinal, irregular longitudinal, oblique, and with/without forming clear/inverted V/U-shaped striae between both setal pairs *e_1_-e_1_* and *f_1_-f_1_*, or posterior to *f_1_-f_1_*, and/or striae forming a diamond pattern between *e_1_-f_2_* area. This subgroup includes ten species, listed below:

*O.gossypii* (Zacher, 1921)

*O.grewiae* Meyer, 1965

*O.intermedius* Meyer, 1964

*O.licinus* Baker & Pritchard, 1960

*O.litchii* Lo & Ho, 1989

*O.matthyssei* Rimando, 1962

*O.randriamasii* Gutierrez, 1967

*O.taiwanicus* Tseng, 1990

*O.trichardti* Meyer, 1974

*O.uruma* Ehara, 1966

### *exsiccator* new species subgroup

**Exemplar species.***Tetranychusexsiccator* Zehntner, 1897: 572.

**Diagnosis (based on female).** More than seven (eight, nine or rarely ten) tactile setae on tibia I, and dorsal hysterosoma with various patterns of striae – longitudinal, irregular longitudinal, oblique and with/without forming clear/inverted V/U-shaped striae restricted to between setae *e_1_-e_1_*, or between and posterior to *f_1_-f_1_*; striae not forming a diamond pattern between these setae. This subgroup comprises of 69 species:

*O.afrasiaticus* (McGregor, 1939)

*O.andrei* Gutierrez, 1966

*O.andropogonearum* Gutierrez, 1969

*O.anneke* Baker & Pritchard, 1962

*O.aquilinus* Meyer, 1974

*O.araneum* Davis, 1968

*O.barbatae* Meyer, 1987

*O.bessardi* Gutierrez, 1966

*O.calicicola* Knihinicki & Flechtmann, 1999

*O.campestris* Meyer, 1987

*O.castrensis* Meyer, 1987

*O.chazeaui* Gutierrez, 1970

*O.dactyloni* Smiley & Baker, 1995

*O.digitatus* Davis, 1966

*O.duncombei* Meyer, 1974

*O.ephamnus* Beard & Walter, 2003

*O.etiennei* Gutierrez, 1982

*O.exsiccator* (Zehntner, 1897)

*O.flexuosus* Beer & Lang, 1958

*O.formosanus* Lo, 1969

*O.gramineus* (McGregor, 1950)

*O.grastis* Meyer, 1974

*O.gratus* Tseng, 1990

*O.grypus* Baker & Pritchard, 1960*

*O.hortulanus* Meyer, 1974

*O.indicus* (Hirst, 1923)

*O.kadarsani* Ehara, 1969

*O.keiferi* Tuttle & Baker, 1968

*O.krantzi* Zaher, Gomaa & El-Enany, 1982

*O.leandrianae* Gutierrez, 1970

*O.manishi* Gupta, 1979

*O.martensis* Meyer, 1974

*O.mcgregori* (Baker & Pritchard, 1953)

*O.menezesi* Flechtmann, 1981

*O.modestus* (Banks, 1900)

*O.mexicanus* (McGregor & Ortega, 1953)

*O.nasutus* Meyer, 1974

*O.nelensis* Meyer, 1974

*O.neoplegas* Meyer, 1964

*O.neopratensis* Meyer, 1974

*O.neotylus* Zeity & Srinivasa, 2016

*O.obliquus* Ehara & Masaki, 2001

*O.ocellatus* Meyer, 1987

*O.oenotherae* Smiley & Baker, 1995

*O.orthius* Rimando, 1962

*O.oryzae* (Hirst, 1926)

*O.palus* Beard, 2008

*O.pennisetum* Meyer, 1974

*O.plegas* Baker & Pritchard, 1960

*O.plicarum* De Leon, 1957

*O.pratensis* (Banks, 1912)*

*O.rubicundus* Ehara, 1971

*O.rusticus* Meyer, 1974

*O.shinkajii* Ehara, 1963

*O.sacchari* (McGregor, 1942)

*O.saccharinus* Baker & Pritchard, 1960

*O.saccharoides* Baker & Tuttle, 1972

*O.sayedi* Zaher, Gomaa & El-Enany, 1982

*O.senegalensis* Gutierrez & Etienne, 1981

*O.simus* Baker & Pritchard, 1960

*O.stickneyi* (McGregor, 1920)

*O.triandrae* Meyer, 1974

*O.turbelli* Beard & Walter, 2003

*O.tylus* Baker & Pritchard, 1960

*O.velascoi* Rimando, 1962

*O.virens* Gutierrez, 1969

*O.waltersi* Meyer, 1987

*O.zanclopes* Beard & Walter, 2003

*O.zeae* (McGregor, 1955)

### Ungrouped *Oligonychus* species

Among 211 *Oligonychus* species, the 17 species listed below were described based on females alone, with the males being unknown in the original and subsequent descriptions (14 of which are also listed as species inquirendae (see further below). Due to the unavailability of critical morphological information regarding the aedeagus shape/orientation, these species could not be assigned to any of the subgenera, species groups or subgroups:

*O.amiensis* Ehara & Gotoh, 2007**

*O.caucasicus* (Reck, 1956)

*O.changi* Tseng, 1980

*O.conostegiae* Tuttle, Baker & Abbatiello, 1974

*O.daleae* Tuttle, Baker & Abbatiello, 1976

*O.jiangxiensis* Ma & Yuan, 1980

*O.longus* Chaudhri, Akbar & Rasool, 1974

*O.mactus* Tseng, 1990

*O.nielseni* Reeves, 1963

*O.picei* (Canestrini, 1889)

*O.primulae* (Oudemans, 1931)

*O.proteae* Meyer & Ryke, 1959

*O.pongami* Sivakumar & Kunchithapatham, 2014

*O.subtropicus* Tseng, 1980

*O.thelytokus* Gutierrez, 1977

*O.tlaxcensis* Tuttle, Baker & Abbatiello, 1976

*O.vazquezae* Estebanes & Baker, 1968

#### ﻿Species inquirendae in the genus *Oligonychus*

The taxonomic identities of 14 *Oligonychus* species are doubtful and require more investigations to clarify their actual status, and are hereby recognized as species inquirendae. The descriptions of these species have been based mainly on the female, and/or do not include important morphological characters of male/female critical for species identification (Canestrini 1889; Oudemans 1931; Zacher 1932; Ugarov and Nikolskii 1937; Reck 1947, 1956; McGregor 1950; McGregor and Ortega 1953; Meyer and Ryke 1959; Reeves 1963; Estebanes and Baker 1968; Ehara 1969; Chaudhri et al. 1974; Tuttle et al. 1974, 1976; Gutierrez 1977; Ma and Yuan 1980; Tseng 1980, 1990; Lo and Ho 1989). A comprehensive revision of these doubtful *Oligonychus* species is necessary to confirm their taxonomic status. Examination of male specimens from the type locality, and detailed re-descriptions of both male and female specimens from the type/topotype material, supported by integrative taxonomic approaches combining morphological and molecular data, would resolve the issue.

##### ﻿1. Oligonychus picei

Taxon classificationAnimaliaTrombidiformesTetranychidae

(Canestrini, 1889)

E7926F18-AA23-51C0-B063-4176ECEE7621

Tetranychus picei Canestrini, 1889: 502.

###### Host and distribution.

*Picea* sp. (Pinaceae); Italy.

###### Remarks.

*Oligonychuspicei* (Canestrini) was described briefly based only on female, male remains unknown in original (Canestrini 1889) and subsequent descriptions (Pritchard and Baker 1955). Although Pritchard and Baker (1955) examined female paratypes, they did not provide a detailed re-description. They mention that it resembles *O.subnudus* (described from USA on *Pinus* sp., Pinaceae), differing by having comparatively longer dorsal setae. The identity of *O.picei* is doubtful until the male and female are comprehensively described from the type host and locality.

##### ﻿2. Oligonychus primulae

Taxon classificationAnimaliaTrombidiformesTetranychidae

(Oudemans, 1931)

BD371A84-47B5-587E-9AEB-63ACFDFB31AF

Paratetranychus primulae Oudemans, 1931: 291.

###### Host and distribution.

*Primulaobconica* (Primulaceae); Netherlands.

###### Remarks.

*Oligonychusprimulae* (Oudemans) was very poorly described using only the female, without illustrations, and the male was unknown in both the original (Oudemans 1931) and subsequent descriptions (Geijsks 1939; Pritchard and Baker 1955). Furthermore, Oudemans (1931) did not compare it specifically with any closely related species, except mentioning the resemblance of its empodium with *O.ununguis* (Jacobi, 1905) (described from Germany on *Piceaabies*, Pinaceae). Pritchard and Baker (1955) confirmed it to be an *Oligonychus*, but highlighted its taxonomic position as doubtful due to the absence of the male. The identity of *O.primulae* is uncertain until the male and female are comprehensively described from the type host and locality.

##### ﻿3. Oligonychus kobachidzei

Taxon classificationAnimaliaTrombidiformesTetranychidae

(Reck, 1947)

1EAD9C23-BAE2-53F9-88B7-55EFE85BC37C

Paratetranychus kobachidzei Reck, 1947: 472.

###### Host and distribution.

*Corylusavellana* (Betulaceae), *Juglansregia* (Juglandaceae), *Platanusoccidentalis*, *P.orientalis* (Platanaceae) and *Ulmus* sp. (Ulmaceae); Armenia, Azerbaijan and, Georgia.

###### Remarks.

*Oligonychuskobachidzei* (Reck) was described from male and female specimens from type host *Platanusoccidentalis* and type locality Georgia; however, the description lacked the key characters necessary for species confirmation (Reck 1947). Moreover, Reck (1947) did not specifically compare it with any other closely related *Oligonychus* species. Although Bagdasarian (1957) re-described the species from other hosts (*Juglansregia* and *Ulmus* sp.) and locality (Armenia), the description still lacked details of the important morphological characters of both sexes. Further, it seems to us, based on the published literature that Bagdasarian (1957) did not observe the types of *O.kobachidzei*, as it necessary for confirming the taxonomic identity of Armenian specimens, when original description of *O.kobachidzei* was poor. Also, the illustration of aedeagus (Bagdasarian 1957) was not clear and did not appear to be in a completely lateral position. The identity of *O.kobachidzei* and its redescription is doubtful until the male and female are comprehensively described from the type host and locality.

##### ﻿4. Oligonychus caucasicus

Taxon classificationAnimaliaTrombidiformesTetranychidae

(Reck, 1956)

699993FF-2960-599B-974A-66782020371F

Paratetranychus caucasicus Reck, 1956: 17.

###### Host and distribution.

*Carpinusbetulus*, *Corylusavellana* (Betulaceae); Georgia.

###### Remarks.

*Oligonychuscaucasicus* (Reck) was briefly described from only the female, and the male was unknown (Reck 1956). Although the description lacked illustrations, it indicated that the species did belong to the genus *Oligonychus* (Reck 1956). However, its species identity remains questionable, because the author neither described the male nor compared it specifically with any closely related species. The taxonomic status of *O.caucasicus* will be resolved after collecting and describing the male and female from the type host and locality.

##### ﻿5. Oligonychus proteae

Taxon classificationAnimaliaTrombidiformesTetranychidae

Meyer & Ryke, 1959

36F5CBAE-352A-5900-ACBC-E9C1FF0AF6E3

Oligonychus proteae Meyer & Ryke, 1959: 344.

###### Host and distribution.

*Proteacoronata* (Proteaceae); South Africa.

###### Remarks.

*Oligonychusproteae* Meyer & Ryke was described from only females, and details of the male were absent in both the original (Meyer and Ryke 1959) and subsequent descriptions (Meyer 1974). The taxonomic status of this species has remained doubtful since it was proposed (Meyer and Ryke 1959; Meyer 1974), and it was excluded from the list of *Oligonychus* species reported from Africa (Meyer 1987). The species identity of *O.proteae* will be resolved after collecting and describing the male from the type host and locality.

##### ﻿6. Oligonychus nielseni

Taxon classificationAnimaliaTrombidiformesTetranychidae

Reeves, 1963

51A80CFB-12D5-56B1-8BD2-54C2EDA7399B

Oligonychus nielseni Reeves, 1963: 57.

###### Host and distribution.

*Pinusstrobus* (Pinaceae); United States.

###### Remarks.

*Oligonychusnielseni* Reeves was described from only females, and details of the male were absent in both the original (Reeves 1963) and subsequent description (Baker and Tuttle 1994). The species was not specifically compared with any other closely related *Oligonychus* species (Reeves 1963). Moreover, variations have been reported in length of dorsocentral setae of the females from different populations, e.g., *c_1_* reaching to bases of *e_1_* (Reeves 1963) or *c_1_* shorter than the interval between *c_1_-e_1_* (Baker and Tuttle 1994). The species identity of *O.nielseni* will be resolved after collecting and describing the male from the type host and locality.

##### ﻿7. Oligonychus longus

Taxon classificationAnimaliaTrombidiformesTetranychidae

Chaudhri, Akbar & Rasool, 1974

E1216E63-7591-5D8D-8422-1906D9DB880E

Oligonychus longus Chaudhri, Akbar & Rasool, 1974: 147.

###### Host and distribution.

Unknown; United States.

###### Remarks.

*Oligonychuslongus* Chaudhri, Akbar & Rasool was briefly described from female specimens only, and the male was unknown (Chaudhri et al. 1974). The species was placed in the subgenus Reckiella, and although the authors did not specifically compare it with any other closely related species of *Oligonychus*, they did mention that based on the pattern of dorsal striae and length of the female body, this species differed from all species in that subgenus. However, the morphological information available for the female clearly indicates that this species does not actually match the subgenus Reckiella diagnosis of that time (e.g., Tuttle and Baker 1968; Chaudhri et al. 1974; Jeppson et al. 1975). The taxonomic identity of *O.longus* is doubtful until the male is collected and described from the type host and locality.

##### ﻿8. Oligonychus conostegiae

Taxon classificationAnimaliaTrombidiformesTetranychidae

Tuttle, Baker & Abbatiello, 1974

3AFC88BE-2958-5847-9198-1D54B1E4972F

Oligonychus conostegia Tuttle, Baker & Abbatiello, 1974: 15.

###### Host and distribution.

*Conostegiaxalapensis* (Melastomataceae); Mexico.

###### Remarks.

*Oligonychusconostegiae* Tuttle, Baker & Abbatiello was briefly described from only females, and details of the male were absent in both the original (Tuttle et al. 1974) and subsequent description (Tuttle et al. 1976). The authors compared it with the female of *O.gambelli* (described from USA on *Quercusgambelii*, Fagaceae), and both species differed based on the often variable number of tactile setae proximal to the proximal duplex on tarsus I (Tuttle et al. 1974). Later, *O.conostegiae* was separated from females of *O.platani* (described from USA on *Platanusoccidentalis*, Platanaceae) in a diagnostic key, using differences in the comparative lengths of the members of the duplex setae (McGregor 1950; Tuttle et al. 1976). These three species are distributed in similar geographical localities (Migeon and Dorkeld 2021), and their separation requires further taxonomic scrutiny. The species identity of *O.conostegiae* will be clear after collecting and describing the male from the type host and locality.

##### ﻿9. Oligonychus daleae

Taxon classificationAnimaliaTrombidiformesTetranychidae

Tuttle, Baker & Abbatiello, 1976

BDB77858-C6FB-552C-88B3-94D9D6A12D97

Oligonychus daleae Tuttle, Baker & Abbatiello, 1976: 86.

###### Host and distribution.

*Dalea* sp. (Leguminosae); Mexico.

###### Remarks.

*Oligonychusdaleae* Tuttle, Baker & Abbatiello was described from females only, and details of the male were unknown (Tuttle et al. 1976). The authors did not specifically compare it with any other closely related species. However, *O.daleae* females were differentiated from the females of *O.propetes* (described from USA on *Quercusalba*) and *O.quercus* (described from Mexico on *Quercus* sp.) in a diagnostic key, using the striae pattern and comparative length of setae on the dorsal hysterosoma (Tuttle et al. 1976). These three species share similar geographical distributions (Migeon and Dorkeld 2021). The species identity of *O.daleae* will be clear after collecting and describing the male from the type host and locality.

##### ﻿10. Oligonychus changi

Taxon classificationAnimaliaTrombidiformesTetranychidae

Tseng 1980

B76580B1-C323-5E1A-8C2C-E2BC3A6D3BB3

Oligonychus changi Tseng, 1980: 152.

###### Host and distribution.

*Pinus* sp. (Pinaceae); Taiwan

###### Remarks.

*Oligonychuschangi* Tseng was poorly described from females only, and details of the male were absent in both the original (Tseng 1980) and subsequent descriptions (Tseng 1990; Lo and Ho 1989). The author did not specifically compare it with any other closely related species. However, *O.changi* females were distinguished from the females of both *O.subtropicus* (another questionable species reported in same paper; described from Taiwan on *Juniperuschinensis*, Cupressaceae) and *O.perditus* (described from Japan on *Juniperuscommunis*) in a diagnostic key, using the pattern of dorsal hysterosomal striae (Tseng 1980, 1990). The species identity of *O.changi* will be clear after collecting and describing the male from the type host and locality.

##### ﻿11. Oligonychus jiangxiensis

Taxon classificationAnimaliaTrombidiformesTetranychidae

Ma & Yuan, 1980

F4A6D8D2-36FF-5FA9-BCB6-7948A94EAF19

Oligonychus jiangxiensis Ma & Yuan, 1980: 43.

###### Host and distribution.

*Cunninghamialanceolate* (Taxodiaceae); China.

###### Remarks.

*Oligonychusjiangxiensis* Ma & Yuan was briefly described from females only without detailed morphological characterization, and details of the male were absent. The authors did not compare it with any other closely distributed or closely related *Oligonychus* species, but instead compared it with *O.endytus* described from the United States on *Quercus* sp. (Fagaceae) (Migeon and Dorkeld 2021). Recently, Li et al. (2019) observed the type material of *O.jiangxiensis*, but did not re-describe or confirm its actual taxonomic status. The species identity of *O.jiangxiensis* will be clear after collecting and describing the male from the type host and locality.

##### ﻿12. Oligonychus subtropicus

Taxon classificationAnimaliaTrombidiformesTetranychidae

Tseng, 1980

846E8D4F-9257-5EFF-B606-D8660AF8F685

Oligonychus subtropicus Tseng, 1980: 147.

###### Host and distribution.

*Juniperuschinensis* (Cupressaceae); Taiwan.

###### Remarks.

*Oligonychussubtropicus* Tseng was described from only females, and details of the male were absent in both the original (Tseng 1980) and subsequent descriptions (Tseng 1990; Lo and Ho 1989). Although the author did not specifically compare it with any closely related species, it was differentiated from *O.perditus* (from Japan on *J.communis*) in a diagnostic key, using slight differences in the pattern of dorsal hysterosomal striae (Tseng 1980, 1990). Moreover, the author did not even compare it with another closely related species, *O.chamaecyparisae* Ma & Yuan (1976) reported from China on *Chamaecyparispisifera* and other Cupressaceae hosts. Bolland et al. (1998) synonymized *O.chamaecyparisae* with *O.perditus* and Migeon and Dorkeld (2021) follow this classification, despite it recently being reinstated as valid species by Li et al. (2019). The species identity of *O.subtropicus* will be clear after collecting and describing the male from the type host and locality.

##### ﻿13. Oligonychus mactus

Taxon classificationAnimaliaTrombidiformesTetranychidae

Tseng, 1990

C8F70965-D55F-5FCB-908A-44C533FBB96F

Oligonychus mactus Tseng, 1990: 146.

###### Host and distribution.

*Pinus* sp. (Pinaceae); Taiwan.

###### Remarks.

*Oligonychusmactus* Tseng was described from females only, and the male is unknown. Tseng differentiated the female from the females of *O.clavatus* (Ehara 1959) (described from Japan on *Pinus* spp.) and *O.subnudus* (McGregor 1950) (from USA on *Pinus* sp.), using the patterns of dorsal hysterosomal striae, the comparative lengths of prodorsal setae and the number of setae on tibia II (Tseng 1990). These three species inhabit *Pinus* spp., but were described from geographically well separated localities (Migeon and Dorkeld 2021). The male of *O.mactus* still needs to be collected and described from the type host and locality.

##### ﻿14. Oligonychus pongami

Taxon classificationAnimaliaTrombidiformesTetranychidae

Sivakumar & Kunchithapatham, 2014

50903194-F21B-57BE-8F32-C47E2B7F0FF6

Oligonychus pongami Sivakumar & Kunchithapatham, 2014: 4113–4117.

###### Host and distribution.

*Pongamiaglabra* (Fabaceae), *Vitisvinifera* (Vitaceae); Coimbatore and Tamil Nadu, India.

###### Remarks.

The description of *Oligonychuspongami* Sivakumar & Kunchithapatham was based on just one morphological character, that the female differs from *O.biharensis* by having longitudinal striations between *e_1_-e_1_* vs. transverse in the later. There are numerous species in the genus *Oligonychus* which have longitudinal striation between setae *e_1_-e_1_*. No details of the male were provided, and the taxonomic identity of *O.pongami* is doubtful until detailed descriptions of the male and female type specimens are provided.

#### ﻿Species complexes in the genus *Oligonychus*

The term species complex, also referred to as sibling or cryptic species complex, is an informal taxonomic term or “open nomenclature qualifier” that is used when two/more morphologically indistinguishable but biologically separate species are present or several distinct species are suspected to exist under one name, which results in the taxonomic uncertainty of a taxon (Sigovini et al. 2016). Species complexes are notoriously difficult to resolve when based on morphology alone (Pritchard and Baker 1955). However, such complex taxonomic issues have been efficiently and effectively addressed in different tetranychid genera, e.g., *Mononychellus* Wainstein, *Oligonychus*, and *Tetranychus* through the combination of morphological, molecular, and biological data (Navajas et al. 1994, 2001; Gotoh et al. 1998, 2007, 2009; Matsuda et al. 2013; Zeity et al. 2017). Therefore, integrative taxonomic approaches are needed to clarify the actual status of all closely related species, and species complexes, in the genus *Oligonychus*.

Within the genus *Oligonychus*, we recognized five new species complexes, viz. the *afrasiaticus* species complex, the *litchi* species complex, the *punicae* species complex, the *plegas* species complex and the *tylus* species complex, along with two previously highlighted complexes, the *sacchari* complex (Khanjani et al. 2018) and the *pratensis* complex (Pritchard and Baker 1955; Meyer 1974; Li et al. 2018). The *punicae* complex is placed in the subgenus Oligonychus, while the other six species complexes are placed in the subgenus Reckiella. The *punicae* species complex includes four morphologically similar *Oligonychus* species, *O.punicae*, *O.mangiferus*, *O.yusti*, and *O.vitis*. The males and females of each of these four species share similar morphology, including a downturned aedeagus, and based on the currently available diagnostics, species of the *punicae* complex are very difficult to distinguish from each other. The *afrasiaticus* complex includes *O.aquilinus*, *O.afrasiaticus*, *O.keiferi*, and *O.menezesi*. The *litchii* complex includes only *O.litchii* and *O.taiwanicus*. The *plegas* complex includes *O.araneum*, *O.orthius*, *O.plegas*, *O.sayedi*, and *O.velascoi*. The *tylus* complex includes *O.etiennei*, *O.senegalensis* and *O.tylus*. Those *Oligonychus* species which belonging to the *afrasiaticus*, *litchii*, *plegas*, and *tylus* complexes can be hardly distinguished using aedeagus shape, number of tactile/sensory setae on tibia I and tactile setae behind to proximal duplex on tarsus I in male, length-width ratio of male/female palp spinneret and comparative length of proximo-ventral spur/main claw of male empodium I. Species that belong to the *sacchari* complex (*O.sacchari* and *O.saccharinus*) and the *pratensis* complex (*O.pratensis*, *O.shinkajii*, and *O.virens*) are difficult discriminate from each other using the available morphological characters because they are variable in their expression.

#### ﻿Presence and absence of para-anal setae in the genus *Oligonychus*

Lindquist (1985) used the form and position of the H setae when determining setal homologies and recognized that both of the para-anal setae *h_2_* and *h_3_* are always present, and that the clunal seta *h_1_* is always absent in the genera *Oligonychus* and *Tetranychus*. This nomenclature was first interpreted by Oudemans (1930) and then later by Pritchard and Baker (1952), and as is followed here.

**Figure 1.**
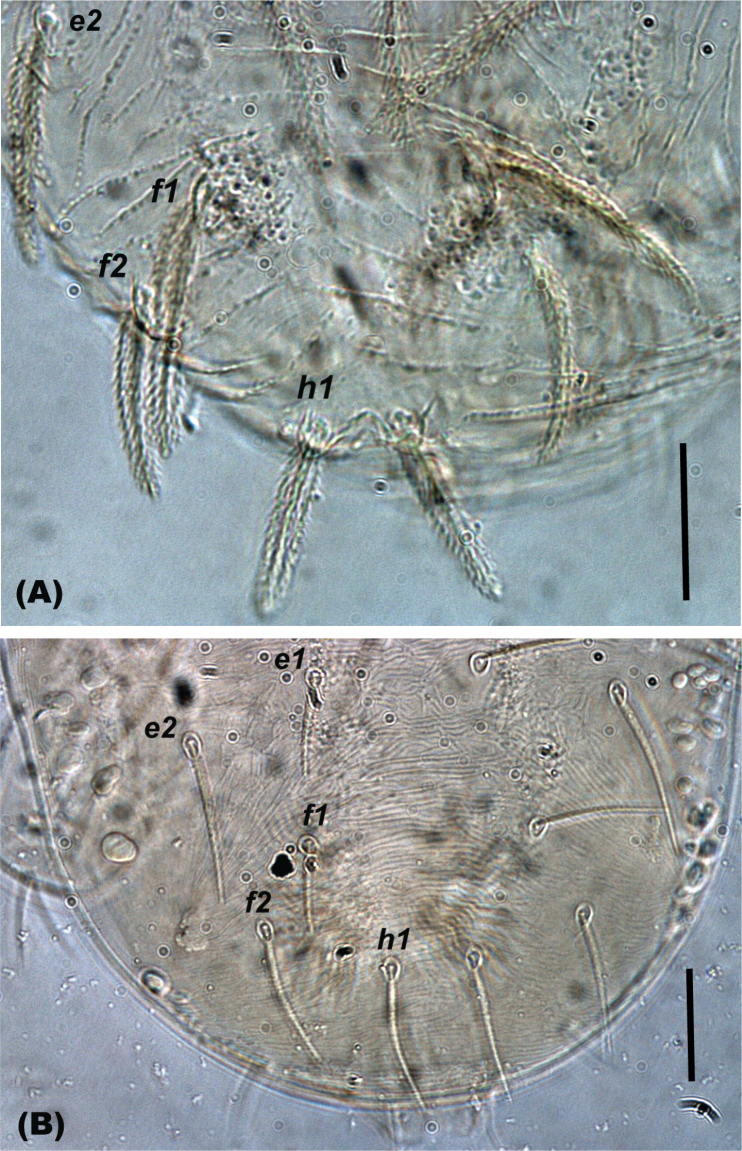
Shape of seta *h_1_* is similar to other dorsal body setae in various genera of the tribe Tetranychini in e.g. **A***Mixonychus* and **B***Schizotetranychus.* Scale bar: 30 μm.

**Figure 2.**
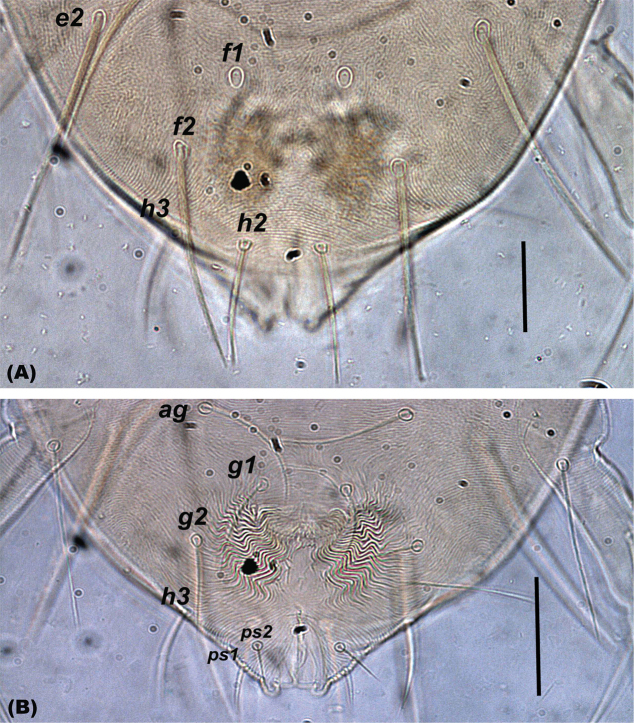
Shape of setae **A***h_2_* and **B***h_3_* are similar to other ventral body setae in all the genera of the tribe Tetranychini in e.g. *Oligonychus.* Scale bar: 30 μm.

**Figure 3.**
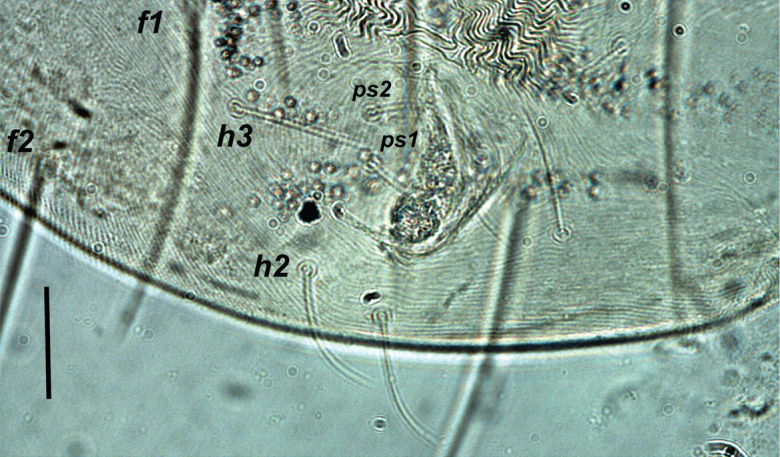
Shape of setae *h_2_* and *h_3_* are similar to other ventral body setae in all the genera of the tribe Tetranychini in e.g *Tetranychus.* Scale bar: 30 μm.

The setal shapes can be helpful when determining the presence or absence (and hence names) of para-anal setae in *Oligonychus* and its closely related genera, as previously highlighted by Lindquist (1985) and Seeman and Beard (2011). Moreover, the form of setae *h_1_* in different genera, for example *Mixonychus* and *Schizotetranychus*, are similar to other dorsal setae (Figs 1A, B), whereas setae *h_2_* and *h_3_* are usually similar in form to the anals, genitals, and other ventral setae, in all Tetranychini genera (Figs 2, 3).

The term “para-anal setae” was introduced by Pritchard and Baker (1955) and can be confusing when interpreting setae in the Tetranychini. So, for practical purposes, the first couplet (page 9, section 2.3) of the diagnostic key to genera of the tribe Tetranychini provided in the world catalogue of spider mites by Bolland et al. (1998) may be interpreted by users as, “3 pairs of *h* setae present, *h_1-3_*” versus “2 pairs of *h* setae present, *h_2-3_* and *h_1_* absent”.

#### ﻿Key to subgenera, species groups, and subgroups of the genus *Oligonychus* Berlese

**Table:** 

1	In lateral view, male aedeagus with shaft bending ventrad	**(subgenus Oligonychus Berlese) 2**
–	In lateral view, male aedeagus with shaft bending dorsad, or shaft initially bending dorsad then sigmoid or curved downward distally	**(subgenus Reckiella Tuttle and Baker) 6**
2	Female with 8 or 9 tactile setae on tibia I	**(*peruvianus* species group) 3**
–	Female with 5, 6 or 7 tactile setae on tibia I	**(*coffeae* new species group) 4**
3	Female with dorsal opisthosomal setae *c_1_* long, reaching well beyond bases of setae *d_1_*	***smithi* new species subgroup**
–	Female with dorsal opisthosomal setae *c_1_* short, not reaching bases of setae *d_1_*, almost one-half to three-quarters as long as the distance between *c_1_-d_1_*	***peruvianus* new species subgroup**
4	Female with dorsal opisthosomal setae *c_1_* short, not reaching bases of *d_1_*, almost one-half to three-quarters as long as the interval to *d_1_*	***subnudus* species subgroup**
–	Female with dorsal opisthosomal setae *c_1_* long, reaching to (sub-equal to the distance between *c_1_-d_1_*) or well beyond bases of setae *d_1_*	**5**
5	Female with 5 or 6 tactile setae on tibia I	***aceris* species subgroup**
–	Female with 7 tactile setae on tibia I	***coffeae* new species subgroup**
6	Female with 5, 6 or 7 tactile setae on tibia I	**(*iseilemae* new species group) 7**
–	Female with 8, 9 or 10 tactile setae on tibia I	**(*exsiccator* new species group) 8**
7	Medial dorsal hysterosomal striae forming a reticulated pattern of irregular, elongate elements in female	***comptus* new species subgroup**
–	Medial dorsal hysterosomal striae without a reticulated pattern in female	***iseilemae* new species subgroup**
8	Female with dorsal hysterosomal striae medially between setae *d_1_-f_2_* typically transverse or wavy transverse, rarely with a mixture of wavy longitudinal and oblique striae posterior to setae *f_1_*	**9**
–	Female with dorsal hysterosomal striae typically longitudinal, irregular longitudinal, oblique, or forming a V/U-shaped pattern, anywhere medially between *d_1_-f_2_* area	**10**
9	Female with 5 or 6 tactile setae on tibia II	***pritchardi* new species subgroup**
–	Female with 7 tactile setae on tibia II	***biharensis* new species subgroup**
10	Female with medial dorsal hysterosomal striae longitudinal, irregular longitudinal, oblique with/without forming a V/U-shaped pattern between setae *e_1_* and *e_1_* and between/posterior to *f_1_* and *f_1_*, and with/without forming a diamond pattern between setal rows E and F (Fig. 4A)	***gossypii* new species subgroup**
–	Female with medial dorsal hysterosomal striae longitudinal (Fig. 4B), irregular longitudinal (Fig. 4C), oblique with/without forming a V/U-shaped pattern (Fig. 4D) between either setae *e_1_* and *e_1_* or between/posterior to *f_1_* and *f_1_*, and not forming a diamond pattern between setal rows E and F	***exsiccator* new species subgroup**

**Figure 4.**
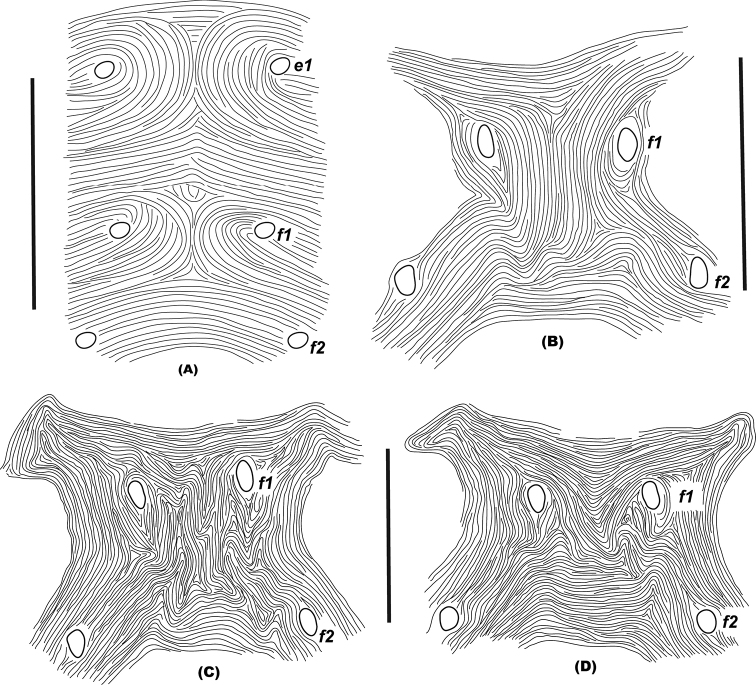
Shape of different striae patterns **A** longitudinal between setae *e_1_-e_1_* and between *f_1_-f_1_*, in *O.randriamasii* Gutierrez (redrawn from original description, Gutierrez 1967) **B** longitudinal between/posterior to setae *f_1_-f_1_*, in *O.orthius* Rimando (redrawn from re-description, Beard et al. 2003) **C** irregular longitudinal between/posterior to setae *f_1_-f_1_***D** oblique with slightly forming V-shaped pattern between setae *f_1_-f_1_*, in *O.turbbelli* Beard and Walter (redrawn from original description, Beard et al. 2003). Scale bars: 50 μm (**B, C, D**); 100 μm (**A**).

## Supplementary Material

XML Treatment for Oligonychus

XML Treatment for Oligonychus picei

XML Treatment for Oligonychus primulae

XML Treatment for Oligonychus kobachidzei

XML Treatment for Oligonychus caucasicus

XML Treatment for Oligonychus proteae

XML Treatment for Oligonychus nielseni

XML Treatment for Oligonychus longus

XML Treatment for Oligonychus conostegiae

XML Treatment for Oligonychus daleae

XML Treatment for Oligonychus changi

XML Treatment for Oligonychus jiangxiensis

XML Treatment for Oligonychus subtropicus

XML Treatment for Oligonychus mactus

XML Treatment for Oligonychus pongami

## Footnotes

We observed a conflict between descriptions and illustrations of O. verduzcoi regarding comparative length of setae c 1 . It is described as shorter in original description by Estebanes and Baker (1968) or reaching to the bases of setae d 1 by Tuttle et al. (1976). We followed the original description (Estebanes and Baker 1968) and assigned this species to the subnudus species subgroup.

Meyer (1974) synonymized O. monsarratiGutierrez (1967) with O. grypus. However, other authors considered it to be a valid species (Bolland et al. 1998; Migeon 2015), and still included in species list on spider mites web (Migeon and Dorkeld 2021). In the present study, we agree with Meyer (1974) by considering it a junior synonym of O. grypus.

Oigonychus pratensis is placed in the exsiccator subgroup based on the reports by various authors (McGregor 1950; Pritchard and Baker 1955; Baker and Pritchard 1962; Estebanes and Baker 1968; Tuttle and Baker 1968; Chaudhri et al. 1974; Tuttle et al. 1976; Baker and Tuttle 1994; Jeppson et al. 1975; Meyer 1987) that the female of this species has longitudinal, irregular longitudinal or oblique striae between setae f 1 -f 1 . Recently, however, Li et al. (2018) reported on a population of O. pratensis with longitudinal striae consistently between both setal pairs e 1 -e 1 and f 1 -f 1 , which is a diagnostic character of the gossypii species subgroup. Because this morphological variation of O. pratensis is consistent and only expressed in China, we believe that this population could actually represent a species in the gossypii subgroup, and that it is not O. pratensis at all. We suggest the use of an integrative taxonomical approach based on both morphological and molecular data to determine its true identity.

The available molecular data for O. amiensis (Matsuda et al. 2012) is proved association with the subgenus Oligonychus. Furthermore, based on female morphology, it belongs to the species group/subgroup coffeae.
